# Coronavirus disease 2019 pneumonia with concomitant spontaneous pneumomediastinum, pneumothorax, and subcutaneous emphysema in a non-ventilated patient, complicated by pulmonary embolism: a case report

**DOI:** 10.1186/s13256-025-05522-6

**Published:** 2025-12-13

**Authors:** Qiuyu Martin Zhu, Amitosh K. Singh, Peter M. Huh, Sandeep Konka

**Affiliations:** 1 Kaiser Permanente Mid-Atlantic States Internal Medicine Residency Program, 655 Watkins Mill Road, Gaithersburg, MD 20879 USA; 2 Pulmonary Medicine/Critical Care Fellowship, Norwalk Hospital, Norwalk, CT 06850 USA; 3 Hospital Medicine, Mid-Atlantic Permanente Medical Group, Rockville, MD 20852 USA

**Keywords:** Pneumomediastinum, Pneumothorax, Subcutaneous emphysema, COVID-19, Case report

## Abstract

**Background:**

Spontaneous pneumomediastinum, pneumothorax, and subcutaneous emphysema are life-threatening complications of coronavirus disease 2019. Concomitant presentation of all three with further complication by pulmonary embolism in non-intubated patients with coronavirus disease 2019 is exceedingly rare and can be devastating.

**Case presentation:**

We present a case of concurrent spontaneous pneumomediastinum, pneumothorax, and subcutaneous emphysema in a non-ventilated, 56-year-old Hispanic male with coronavirus disease 2019 pneumonia that was further complicated by extensive pulmonary embolism. In addition to treatment of coronavirus disease 2019 pneumonia, the patient was treated with an innovative strategy by creating a blowhole incision over the chest wall connected to vacuum-assisted closure therapy resulting in rapid clinical improvement. He was also started on systemic anticoagulation therapy for pulmonary embolism. This patient was eventually discharged home in stable condition.

**Conclusion:**

Our case highlights the critical importance of maintaining clinical vigilance of spontaneous pneumothorax and pneumomediastinum, even in patients not undergoing mechanical ventilation. In addition, it demonstrates the utility of vacuum-assisted closure therapy in treating concurrent pneumothorax and subcutaneous emphysema.

## Background

At the end of 2019, a novel coronavirus was identified as the cause of a cluster of pneumonia cases in Wuhan, China, which was later designated as severe acute respiratory syndrome coronavirus 2 (SARS-CoV-2). In February 2020, the World Health Organization designated the disease coronavirus disease 2019 (COVID-19) [1].

The pulmonary symptoms of COVID-19 range from asymptomatic infections, to mild disease (no pneumonia or mild pneumonia), to severe or critical disease (for example, with dyspnea, hypoxia, or more than 50% lung involvement on imaging) that may require mechanical ventilation, with an overall mortality of 5% [2].

Here, we present a rare case of concomitant spontaneous pneumomediastinum, pneumothorax, and subcutaneous emphysema in a non-ventilated patient with COVID-19 pneumonia, complicated by extensive pulmonary embolism (PE). The patient achieved a favorable outcome through prompt clinical recognition and innovative management using vacuum-assisted closure (VAC) therapy. Given the potentially high mortality rate associated with these complications, our case offers a practical example that can help guide the future management of similar cases.

## Case presentation

A 56-year-old Hispanic male with a history of untreated type 2 diabetes (hemoglobin A1c 12.2%), hypertension, and dyslipidemia presented to the emergency department with progressive dyspnea and hypoxemia. He had been diagnosed with COVID-19 infection 4 days earlier and had been managing his symptoms of fever and sore throat at home. The patient received the Johnson and Johnson SARS-CoV-2 vaccine 4 months earlier. He had no prior lung pathology but was a former smoker (1.25 pack-years), who quit 11 years ago. He denied alcohol or recreational drug use.

On arrival, the patient complained of generalized weakness and intractable hiccups, stating he was so weak that he could not walk at home. His hiccups were persistent and sometimes caused dyspnea. He denied cough, nausea, vomiting, chest pain, or diarrhea. Physical examination revealed fatigue and discomfort due to hiccups. He was afebrile, with tachycardia (pulse 116 beats per minute), tachypnea (respiration 22 breaths per minute), and hypoxia (oxygen saturation of 86% on room air, improving to 93% on 3 L/minute oxygen by nasal cannula). Lung auscultation revealed bilateral rhonchi without wheezing or rales.

Given concern for diaphragmatic irritation secondary to PE causing hiccups, a chest computed tomography (CT) angiography was performed, showing bilateral peripheral ground-glass infiltrates but no evidence of PE. Electrocardiogram (EKG) showed sinus tachycardia of 108 beats per minute, left axis deviation, large P waves, T wave inversions in V1, and no acute ischemic changes. Laboratory studies revealed thrombocytopenia (88 × 10^9^/L), hyponatremia (128 mmol/L), and hyperglycemia (17.9 mmol/L). Remaining labs were otherwise normal, including white blood cell count (6.6 × 10^9^/L), hepatic function, and negative high-sensitivity troponin and creatine kinase. Inflammation markers were elevated, including erythrocyte sedimentation rate (63 mm/hour), ferritin (3118.20 ng/mL), high-sensitivity C-reactive protein (> 200.00 mg/dL), D-dimer (305 ng/mL DDU), and lactate (2.50 mmol/L). The patient received dexamethasone, remdesivir, and tocilizumab. His hiccups improved with chlorpromazine, famotidine, and sucralfate. He received supplemental oxygen via nasal cannula and never required positive-pressure devices.

**Fig. 1:**
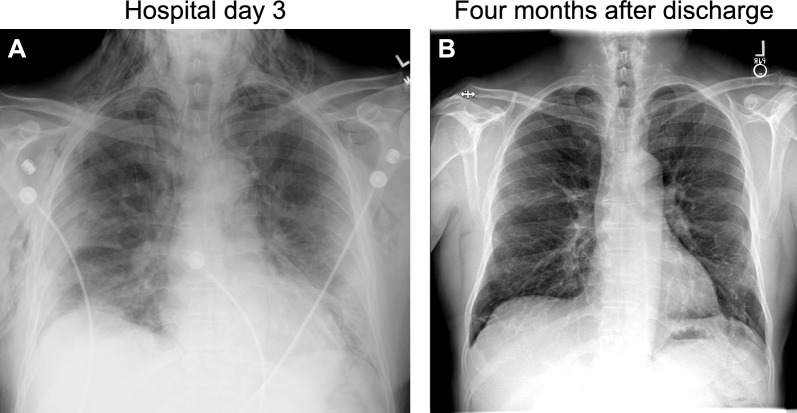
Chest radiography on hospital day 3 (**A**) and at 4 months after discharge (**B**). (**A**) The radiography was obtained on hospital day 3 after the patient developed a “popping sensation” in the neck, showing diffuse bilateral lung infiltrates and extensive subcutaneous emphysema. There was widening of the mediastinum consistent with pneumomediastinum, although it could have been positional. (**B**) At 4-month follow up, the radiography showed significant improvement of bilateral lung infiltrates and complete resolution of subcutaneous edema

**Fig. 2:**
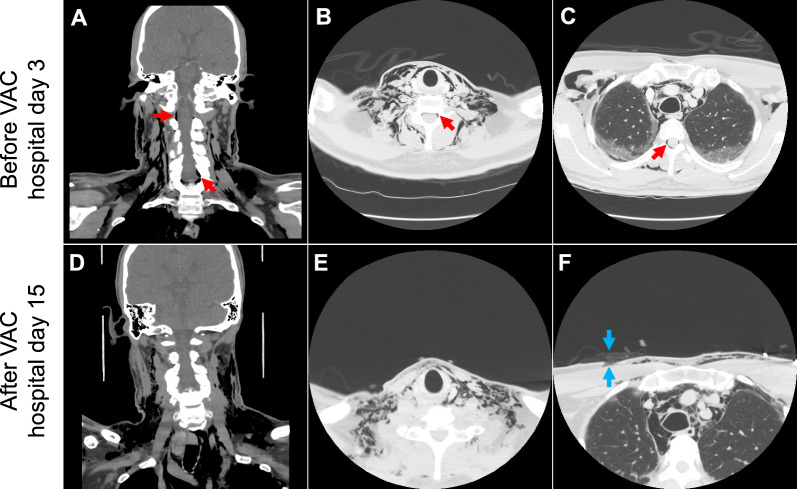
Computed tomography of the neck obtained before (**A**–**C**, on hospital day 3) and after vacuum-assisted closure therapy (**D**–**F**, on hospital day 15). (**A**–**C**) The images were obtained immediately after the patient developed a “popping sensation” in the neck, showing extensive subcutaneous emphysema tracking along the fat planes in the bilateral neck and chest. There was extensive pneumomediastinum and right apical pneumothorax. Air was also in the epidural space (red arrows). (**D**–**F**) After VAC therapy, the overall volume of superficial emphysema throughout the neck decreased. The right anterior chest blowhole incision is seen connected to topical vacuum-assisted closure dressing (blue arrows). (**A** and **D**), coronal section; (**B, C, E, and F**), axial section

**Fig. 3:**
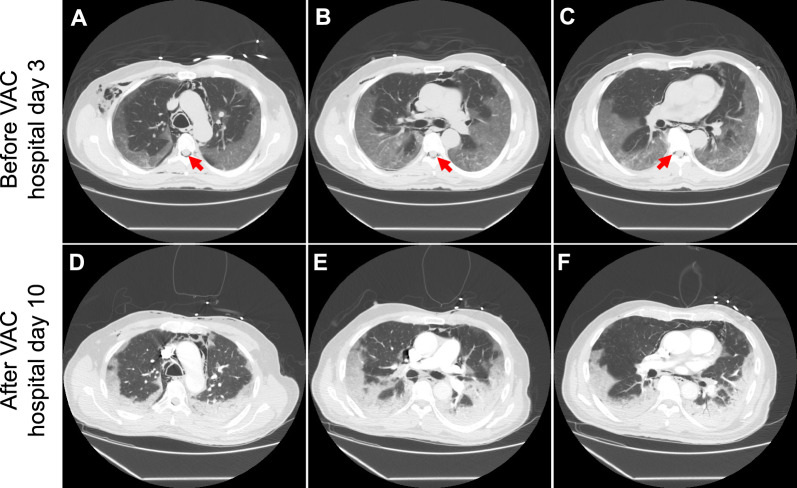
Computed tomography of the chest obtained before (**A**–**C**, on hospital day 3) and after vacuum-assisted closure therapy (**D**–**F**, on hospital day 10). (**A**–**C**) The images were obtained on the day when the patient developed chest pain and a “popping sensation” in the neck, showing extensive subcutaneous emphysema and pneumomediastinum. Gas tracks along the right pleural space. Epidural gas is also seen within the thoracic spine (red arrows). (**D**–**F**) The images were obtained on hospital day 10 to rule out pulmonary embolism when the patient developed worsening hypoxia, while on vacuum-assisted closure therapy. The overall decrease in the volume of soft tissue emphysema can be appreciated compared with (**A–C**). Significant bilateral consolidation compatible with coronavirus disease 2019 infection appears similar on the two sets of images

On hospital day 3, the patient complained of sudden-onset neck and chest pain with a popping sensation in his right neck after a coughing spell. Physical examination showed mild swelling along the right neck border without crepitus or respiratory distress. A prompt chest radiography revealed diffuse bilateral lung infiltrates and extensive subcutaneous emphysema (Fig. 1A). CT of the neck and chest showed extensive pneumomediastinum, small right apical pneumothorax, bilateral subcutaneous emphysema extending into the neck, and epidural air (Figs. 2 and 3). Shortly after the imaging, the patient became tachycardic and tachypneic, requiring supplemental oxygen with a non-rebreather mask. Physical examination showed subcostal retractions and crepitus on the upper chest wall and neck. Emergency treatment was initiated by thoracic surgery and intensive care unit (ICU) teams at the bedside via a blowhole incision over the right anterior chest wall connected to a subcutaneous VAC device set at −125 mmHg negative pressure, resulting in immediate clinical improvement. The patient quickly transitioned back to nasal cannula and felt much better.

**Fig. 4:**
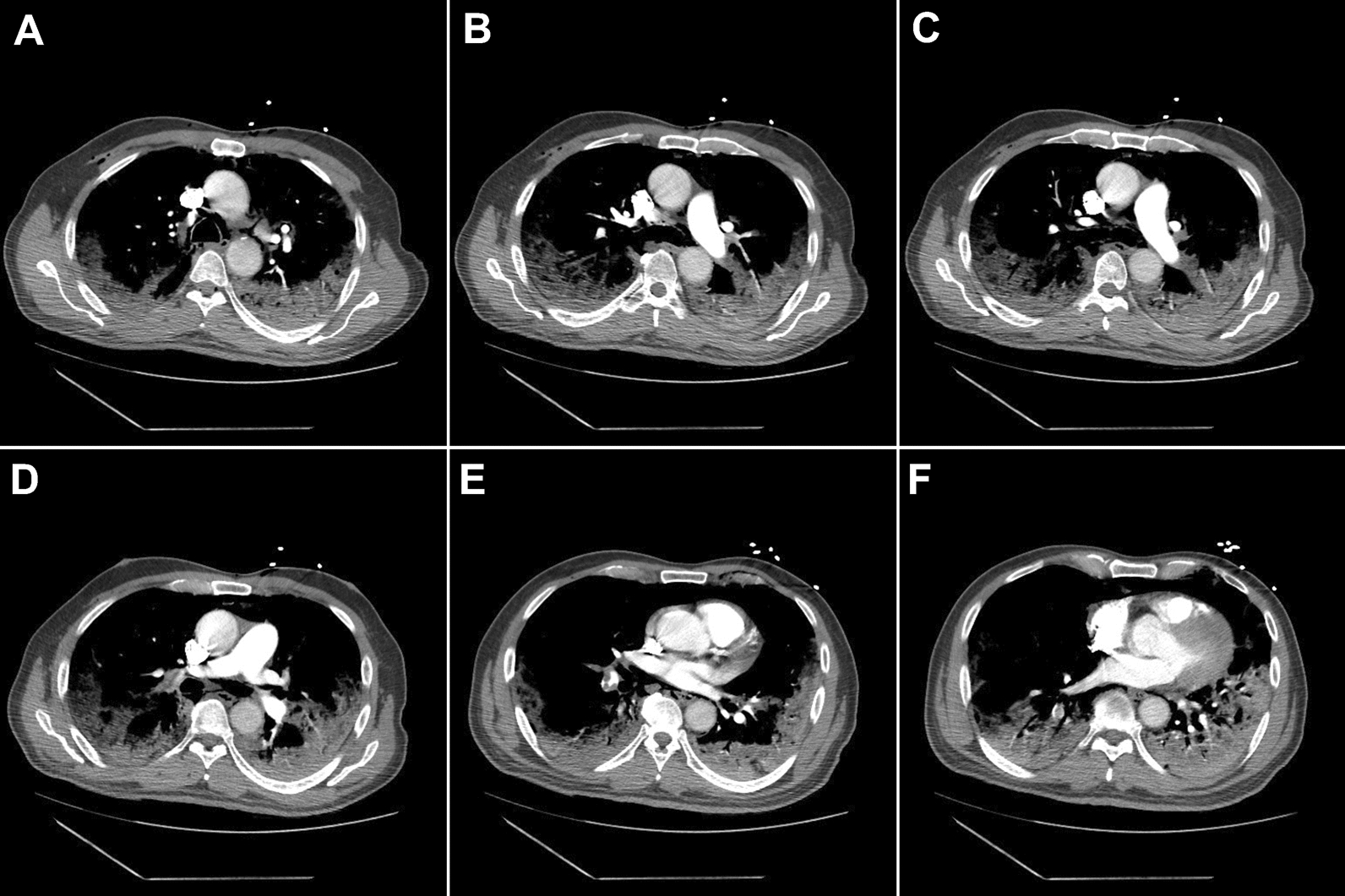
Computed tomography angiography of the chest obtained on hospital day 10. Through (**A**–**F**), there are proximal and peripheral filling defects in pulmonary arteries supplying all three lung fields on the right and peripheral branches on the left. There was no evidence of right heart strain. Significant bilateral pneumonia and pneumomediastinum were better visualized in the lung window shown in Fig. 3

On hospital day 10, the patient developed worsening hypoxia, requiring high-flow nasal cannula. CT angiography showed PE involving all proximal right lung fields and a few peripheral fields on the left (Fig. 4). He started anticoagulation therapy with subcutaneous enoxaparin.

By hospital day 16, his oxygen requirement decreased to 3 L/minute by nasal cannula. The pneumomediastinum, pneumothorax, and subcutaneous emphysema reduced with the VAC therapy (Figs. 2 and 3) and remained stable after VAC removal. He was discharged with dabigatran, tapering steroids, and home oxygen.

The patient completed 3 months of dabigatran, underwent home physical rehabilitation, and showed significant clinical improvement. At the 3-month follow-up, he had weaned off supplemental oxygen, returned to normal activities and work, and could walk 10,000 steps daily. Repeat chest radiography showed complete resolution of the pneumothorax and significant improvement in lung opacity (Fig. 1B).

## Discussion

The clinical presentation of COVID-19 is highly variable, and our understanding continues to evolve. Concomitant spontaneous pneumomediastinum, pneumothorax, and subcutaneous emphysema are rare, accounting for less than 1% of COVID-19 complications [3, 4]. However, their concurrent presentation results in a high mortality rate of up to 26% [5], necessitating high clinical vigilance even in patients who are not mechanically ventilated.

A spontaneous pneumothorax refers to gas in the pleural space without an external event. It is classified as primary (no underlying lung disease) and secondary (complication of existing lung disease). A large pneumothorax may be accompanied by subcutaneous emphysema. Nearly all lung diseases can lead to secondary spontaneous pneumothorax, with chronic obstructive pulmonary disease being the most common cause, followed by tuberculosis in endemic areas [6]. The first occurrence of spontaneous pneumothorax in patients with COVID-19 was reported in Wuhan, China, found in 5.9% of intubated patients [7]. Subsequent case series later reported varying incidences in patients with COVID-19 [8–10]. The presumed pathophysiological mechanism involves direct invasion and necrosis of lung tissue, including the pleura, by SARS-CoV-2, causing air leak due to extensive diffuse alveolar damage, followed by alveolar rupture [11]. The risk correlates with the severity of the infection [12–16].

Pneumomediastinum can occur with or without pneumothorax and is a marker of severe COVID-19 infection. A sevenfold increase in pneumomediastinum incidence has been associated with COVID-19 compared with non-SARS-CoV-2 infection [17]. In the UK, pneumomediastinum was identified in 0.64% of COVID-19 inpatients, with a 120-day mortality of 51.7% [18]. A Romanian study reported an incidence of 0.86% among COVID-19 inpatients [19]. About half of these cases were not associated with mechanical ventilation at the time of diagnosis [18–20].

The risk factors for spontaneous pneumothorax or pneumomediastinum in COVID-19 are not fully understood. Although more than half of these complications were not linked to positive-pressure ventilation, barotrauma was commonly attributed as the primary cause and a significant predictor of mortality [18]. The incidence of pneumothorax is higher in males [21]. High serum lactate dehydrogenase levels are associated with pneumomediastinum [5]. History of smoking, known pulmonary pathology, and viral load do not appear to increase the risk [4, 6].

Our patient initially presented with intractable hiccups, a known risk factor for spontaneous pneumomediastinum [22, 23] and an atypical presentation of COVID-19 [24, 25]. His pneumothorax and pneumomediastinum developed after a coughing spell. Severe and persistent hiccups, forceful coughing, vomiting, sneezing, excessive straining, or asthma exacerbation can elevate intrathoracic pressure, triggering pneumomediastinum by the Macklin effect. The co-occurrence of hiccups and acute hyponatremia, as seen in our patient, has been reported as a unique manifestation of COVID-19 [26, 27]. Intractable hiccups in COVID-19 should be promptly addressed to reduce the risk of pneumothorax or pneumomediastinum.

No guidelines exist for managing spontaneous pneumothorax or pneumomediastinum in patients with COVID-19. High clinical vigilance is crucial for early diagnosis in any patient with COVID-19 with sudden onset chest pain or respiratory distress. Management options, based on etiology, for spontaneous pneumothorax and pneumomediastinum in COVID-19 infection, have been recently reviewed elsewhere [28, 29]. Briefly, for unstable patients, needle decompression or emergent chest tube placement should be performed. For stable patients, supplemental oxygen and removal of air by chest tube or catheter thoracostomy are recommended. Conservative measures such as oxygen and observation may be reasonable for mildly symptomatic patients. It should be emphasized that high-flow nasal cannula and noninvasive ventilation should be avoided, since these therapies deliver positive pressure to the airway that could potentially perpetuate the air leak. Chlorpromazine has been effective in improving hiccups associated with COVID-19 in a period of 10 hours [30, 31].

Our patient benefited from a blowhole incision connected to VAC, resulting in rapid clinical improvement. This technique was first reported in 1992 for subcutaneous emphysema, where an infraclavicular blowhole incision was made under local anesthesia that could immediately decompress subcutaneous emphysema [32]. However, without negative-pressure therapy, blowholes can collapse owing to clots, tissue recoil, and scarring [33]. Later, negative-pressure wound therapy, known as VAC, introduced by Argenta and Morykwasin in 1997, was leveraged for its capacity to pull air and fluid out of wounds, enhancing healing. VAC placement for subcutaneous emphysema has several advantages. First, VAC is a rapid, minimally invasive therapy that can be performed at the bedside with local anesthesia. Second, VAC allows for constant air aspiration, lung expansion, and early apposition of the pleura surfaces, leading to rapid improvement of respiratory improvement. Previous cases showed subcutaneous emphysema can resolve within 2–4 days of VAC therapy [34] compared with several weeks with observation alone. Third, VAC also improves blood flow, reduces edema, promotes granulation tissue formation, and decreases inflammatory mediators.

Benefits of VAC therapy should be viewed cautiously owing to the paucity of reports, and adverse effects should be considered. Pain at the wound site is the most common complication of VAC therapy, which is usually controlled by adjusting the pressure [34]. Other less common adverse effects for VAC treatment, usually inappropriately managed system, include bleeding, infection, tissue damage, allergic reaction to materials, fistula formation, wound dehiscence, and reopening of the wound [35–37].

The bilateral PE in our patient was expected, given the hypercoagulable state associated with COVID-19 that can cause thromboembolism [38]. However, the concomitant occurrence of pneumomediastinum, pneumothorax, subcutaneous emphysema, and extensive PE in COVID-19 is exceedingly rare, with only one previously reported case, which was fatal [39].

The prognosis of concomitant spontaneous pneumothorax and pneumomediastinum in patients with COVID-19 is poor, with a mortality rate of up to 26% [5]. Factors associated with higher mortality include mechanical ventilation, older age, and diabetes mellitus [18, 21]. One study indicated a higher mortality among females, patients with body mass index ≥ 30 kg/m^2^, those who were intubated, and those with pre-existing lung disease [20].

## Conclusion

This case highlights the risk for developing spontaneous pneumothorax and pneumomediastinum in patients with COVID-19 pneumonia, even in the absence of positive-pressure ventilation. Reversible risk factors, such as maneuvers that elevate intrathoracic pressure, should be closely monitored and promptly addressed to prevent severe respiratory complications. A high level of clinical vigilance and early imaging are essential for early diagnosis. Negative-pressure therapy, such as VAC, can be promptly implemented at bedside, leading to a favorable clinical outcome.

## Data Availability

All data generated or analyzed during this study are included in this published article.

## Abbreviations

COVID-19: Coronavirus disease 2019
PE: Pulmonary embolism
VAC: Vacuum-assisted closure
SARS-CoV-2: Severe acute respiratory syndrome coronavirus 2
CT: Computed tomography

## References

[1] Eurosurveillance editorial: Note from the editors: World Health Organization declares novel coronavirus (2019-nCoV) sixth public health emergency of international concern. Euro Surveill 2020; 25. 10.2807/1560-7917.ES.2020.25.5.200131e PMC7014669 32019636

[2] Stokes EK, Zambrano LD, Anderson KN, Marder EP, Raz KM, El Burai Felix S, Tie Y, Fullerton KE. Coronavirus disease 2019 case surveillance - United States, January 22-May 30, 2020. MMWR Morb Mortal Wkly Rep. 2020;69:759–65. 32555134 10.15585/mmwr.mm6924e2 PMC7302472

[3] Miro O, Llorens P, Jimenez S, Pinera P, Burillo-Putze G, Martin A, Martin-Sanchez FJ, Garcia-Lamberetchs EJ, Jacob J, Alquezar-Arbe A, *et al*. Frequency, risk factors, clinical characteristics, and outcomes of spontaneous pneumothorax in patients with coronavirus disease 2019: a case-control, emergency medicine-based multicenter study. Chest. 2021;159:1241–55. 33227276 10.1016/j.chest.2020.11.013 PMC7678420

[4] Cut TG, Tudoran C, Lazureanu VE, Marinescu AR, Dumache R, Tudoran M. Spontaneous pneumomediastinum, pneumothorax, pneumopericardium and subcutaneous emphysema-not so uncommon complications in patients with COVID-19 pulmonary infection-a series of cases. J Clin Med. 2021;10: . 33805118 10.3390/jcm10071346 PMC8036962

[5] Elhakim TS, Abdul HS, Pelaez Romero C, Rodriguez-Fuentes Y. Spontaneous pneumomediastinum, pneumothorax and subcutaneous emphysema in COVID-19 pneumonia: a rare case and literature review. BMJ Case Rep. 2020. 10.1136/bcr-2020-239489. 33310838 10.1136/bcr-2020-239489 PMC7735137

[6] Hallifax RJ, Goldacre R, Landray MJ, Rahman NM, Goldacre MJ. Trends in the incidence and recurrence of inpatient-treated spontaneous pneumothorax, 1968–2016. JAMA. 2018;320:1471–80. 30304427 10.1001/jama.2018.14299 PMC6233798

[7] Yao W, Wang T, Jiang B, Gao F, Wang L, Zheng H, Xiao W, Yao S, Mei W, Chen X, *et al*. Emergency tracheal intubation in 202 patients with COVID-19 in Wuhan, China: lessons learnt and international expert recommendations. Br J Anaesth. 2020;125:e28–37. 32312571 10.1016/j.bja.2020.03.026 PMC7151238

[8] Ucpinar BA, Sahin C, Yanc U. Spontaneous pneumothorax and subcutaneous emphysema in COVID-19 patient: case report. J Infect Public Health. 2020;13:887–9. 32475804 10.1016/j.jiph.2020.05.012 PMC7247978

[9] Quincho-Lopez A, Quincho-Lopez DL, Hurtado-Medina FD. Case report: pneumothorax and pneumomediastinum as uncommon complications of COVID-19 pneumonia-Literature review. Am J Trop Med Hyg. 2020;103:1170–6. 32705978 10.4269/ajtmh.20-0815 PMC7470555

[10] Mohan V, Tauseen RA. Spontaneous pneumomediastinum in COVID-19. BMJ Case Rep. 2020. 10.1136/bcr-2020-236519. 32457032 10.1136/bcr-2020-236519 PMC7252963

[11] Fox SE, Akmatbekov A, Harbert JL, Li G, Quincy Brown J, Vander Heide RS. Pulmonary and cardiac pathology in African American patients with COVID-19: an autopsy series from New Orleans. Lancet Respir Med. 2020;8:681–6. 32473124 10.1016/S2213-2600(20)30243-5 PMC7255143

[12] McGuinness G, Zhan C, Rosenberg N, Azour L, Wickstrom M, Mason DM, Thomas KM, Moore WH. Increased incidence of barotrauma in patients with COVID-19 on invasive mechanical ventilation. Radiology. 2020;297:E252–62. 32614258 10.1148/radiol.2020202352 PMC7336751

[13] Udi J, Lang CN, Zotzmann V, Krueger K, Fluegler A, Bamberg F, Bode C, Duerschmied D, Wengenmayer T, Staudacher DL. Incidence of barotrauma in patients with COVID-19 pneumonia during prolonged invasive mechanical ventilation - A case-control study. J Intensive Care Med. 2021;36:477–83. 32959730 10.1177/0885066620954364

[14] Kahn MR, Watson RL, Thetford JT, Wong JI, Kamangar N. High incidence of barotrauma in patients with severe coronavirus disease 2019. J Intensive Care Med. 2021;36:646–54. 33722090 10.1177/0885066621989959 PMC7967021

[15] Rajdev K, Spanel AJ, McMillan S, Lahan S, Boer B, Birge J, Thi M. Pulmonary barotrauma in COVID-19 patients with ARDS on invasive and non-invasive positive pressure ventilation. J Intensive Care Med. 2021;36:1013–7. 34013825 10.1177/08850666211019719

[16] Gupta VK, Alkandari BM, Mohammed W, Tobar AM, Abdelmohsen MA. Ventilator associated lung injury in severe COVID-19 pneumonia patients - case reports: ventilator associated lung injury in COVID-19. Eur J Radiol Open. 2021;8: 100310. 33364262 10.1016/j.ejro.2020.100310 PMC7750144

[17] Lemmers DHL, Abu Hilal M, Bna C, Prezioso C, Cavallo E, Nencini N, Crisci S, Fusina F, Natalini G. Pneumomediastinum and subcutaneous emphysema in COVID-19: barotrauma or lung frailty? ERJ Open Res. 2020. 10.1183/23120541.00385-2020. 33257914 10.1183/23120541.00385-2020 PMC7537408

[18] Melhorn J, Achaiah A, Conway FM, Thompson EMF, Skyllberg EW, Durrant J, Hasan NA, Madani Y, Naran P, Vijayakumar B, *et al*. Pneumomediastinum in COVID-19: a phenotype of severe COVID-19 pneumonitis? The results of the United Kingdom (POETIC) survey. Eur Respir J. 2022;60:3. 10.1183/13993003.02522-2021 PMC8832377 35144988

[19] Cristea AM, Zaharia DC, Leu S, Bogdan MA. Complications during hospitalization in patients with SARS-CoV-2 pneumonia in a Romanian pulmonary center. Cureus. 2023;15: e33882. 36819389 10.7759/cureus.33882 PMC9934938

[20] Gulati U, Medeiros C, Nanduri A, Kanoff J, Zarbiv S, Bonk M, Green A. Understanding pneumomediastinum as a complication in patients with COVID-19: a case series. J Invest Med High Impact Case Rep. 2022;10: 23247096221127117. 10.1177/23247096221127117 PMC9490390 36125171

[21] Martinelli AW, Ingle T, Newman J, Nadeem I, Jackson K, Lane ND, Melhorn J, Davies HE, Rostron AJ, Adeni A, *et al*. COVID-19 and pneumothorax: a multicentre retrospective case series. Eur Respir J. 2020. 10.1183/13993003.02697-2020. 32907891 10.1183/13993003.02697-2020 PMC7487269

[22] Na SJ, Lee SI, Chung TS, Choi YC, Lee KY. Pneumomediastinum due to intractable hiccup as the presenting symptom of multiple sclerosis. Yonsei Med J. 2005;46:292–5. 15861505 10.3349/ymj.2005.46.2.292 PMC2823028

[23] Patino-Galeana JL, Reveles-Garcia MP, Nino-Rodriguez CM, Suarez-Ramirez K, Renteria-Solis IE. Spontaneous pneumomediastinum associated with severe and persistent episode of hiccups. Bol Med Hosp Infant Mex. 2021;78:485–8. 34571523 10.24875/BMHIM.20000332

[24] Portela-Sanchez S, Sanchez-Soblechero A, Melgarejo Otalora PJ, Rodriguez Lopez A, Velilla Alonso G, Palacios-Mendoza MA, Catedra Carame C, Amaya Pascasio L, Mas Serrano M, Massot-Tarrus A, *et al*. Neurological complications of COVID-19 in hospitalized patients: the registry of a neurology department in the first wave of the pandemic. Eur J Neurol. 2021;28:3339–47. 33474816 10.1111/ene.14748 PMC8013314

[25] Nakaya A, Ogura E, Katayama Y, Yoshii M, Yoshino E, Hozumi K, Tago S, Teranishi Y, Minamibashi Y, Harada M, *et al*. Hiccups as a specific neurological manifestation in males with COVID-19. IDCases. 2021;26: e01330. 34777996 10.1016/j.idcr.2021.e01330 PMC8577838

[26] Pandey PK, Pandey D, Andrews R. Hiccups and acute symptomatic hyponatremia: a rare manifestation of COVID-19. Cureus. 2022;14: e24090. 35573499 10.7759/cureus.24090 PMC9106551

[27] Sangamesh S, Gosavi S, Shastry S, Johny SM. Hiccups and hyponatremia: unusual co-presentation in COVID-19. J Family Med Prim Care. 2021;10:1040–3. 34041119 10.4103/jfmpc.jfmpc_1582_20 PMC8138369

[28] Ganessane E, Devendiran A, Ramesh S, Uthayakumar A, Chandrasekar V, Sadasivam AS, Nathan B, Ayyan M. Pneumomediastinum in COVID-19 disease: clinical review with emphasis on emergency management. J Am Coll Emerg Physicians Open. 2023;4: e12935. 37056716 10.1002/emp2.12935 PMC10086517

[29] Sklienka P, Frelich M, Bursa F. Patient self-inflicted lung injury-a narrative review of pathophysiology, early recognition, and management options. J Pers Med. 2023;13:593. 37108979 10.3390/jpm13040593 PMC10146629

[30] Sene DR, Watashi DM, Bilitardo IO, Moreno CEC, Moreno MFF. COVID-19 presenting as persistent hiccups: a case report. Rev Inst Med Trop Sao Paulo. 2021;63: e62. 34378765 10.1590/S1678-9946202163062 PMC8357301

[31] Ikitimur H, Borku Uysal B, Ikitimur B, Umihanic S, Smajic J, Jahic R, Olcay A. Case report: two cases of persistent hiccups complicating COVID-19. Am J Trop Med Hyg. 2021;104:1713–5. 33793414 10.4269/ajtmh.21-0190 PMC8103448

[32] Herlan DB, Landreneau RJ, Ferson PF. Massive spontaneous subcutaneous emphysema. Acute management with infraclavicular “blow holes.” Chest. 1992;102:503–5. 1340766 10.1378/chest.102.2.503

[33] Sciortino CM, Mundinger GS, Kuwayama DP, Yang SC, Sussman MS. Case report: treatment of severe subcutaneous emphysema with a negative pressure wound therapy dressing. Eplasty. 2009;9: e1. 19198645 PMC2627309

[34] Byun CS, Choi JH, Hwang JJ, Kim DH, Cho HM, Seok JP. Vacuum-assisted closure therapy as an alternative treatment of subcutaneous emphysema. Korean J Thorac Cardiovasc Surg. 2013;46:383–7. 24175278 10.5090/kjtcs.2013.46.5.383 PMC3810565

[35] Argenta LC, Morykwas MJ. Vacuum-assisted closure: a new method for wound control and treatment: clinical experience. Ann Plast Surg. 1997;38:563–76. 9188971

[36] Moues CM, Heule F, Hovius SE. A review of topical negative pressure therapy in wound healing: sufficient evidence? Am J Surg. 2011;201:544–56. 21421104 10.1016/j.amjsurg.2010.04.029

[37] Vikatmaa P, Juutilainen V, Kuukasjarvi P, Malmivaara A. Negative pressure wound therapy: a systematic review on effectiveness and safety. Eur J Vasc Endovasc Surg. 2008;36:438–48. 18675559 10.1016/j.ejvs.2008.06.010

[38] Lorenzo C, Francesca B, Francesco P, Elena C, Luca S, Paolo S. Acute pulmonary embolism in COVID-19 related hypercoagulability. J Thromb Thrombolysis. 2020;50:223–6. 32474757 10.1007/s11239-020-02160-1 PMC7260472

[39] Belarbi Z, Brem FL, Nasri S, Imane S, Noha EO. An uncommon presentation of COVID-19: concomitant acute pulmonary embolism, spontaneous tension pneumothorax, pneumomediastinum and subcutaneous emphysema (a case report). Pan Afr Med J. 2021;39:26. 34394817 10.11604/pamj.2021.39.26.29178 PMC8348285

